# Supplementary material for: Learning curve and surgical outcome of robotic assisted colorectal surgery with ERAS program
Source: Sci Rep. 2022 Nov 29;12:20566. doi: 10.1038/s41598-022-24665-w (PMC9709162; doi:10.1038/s41598-022-24665-w)
Supplement: Supplementary file 1 — Supplementary Information 1. [file 41598_2022_24665_MOESM1_ESM.zip › LSC Raw data-Ñ[▒K/105/H_ñw╝╨░O▒Kñσ.pdf]

正式摘要(電子簽章：000000203924837)		
W55 / W55 ☐英文標題(注意:無英文姓名-請補病人基本檔!)	☐【讀取】憑證	

臺中榮民總醫院

出院病歷摘要

轉入號 : 轉出號 :

[redacted]

**(8)**流水編號	
**(9)**入院日期	109/11/19 (2020) 直腸外科 W55 - 272
**(10)**轉科日期	
**(11)**出院日期	109/11/25 (2020) 住院天數計 6 日

診斷

- 入院 : 1. S Colon Cancer, cT1NxM0(SM invasion 170 um), s/p EMR on 2019/12/24
  2. HP infection s/p eradication (20+ years ago)
- 出院 : 1. S Colon Cancer, cT1NxM0(SM invasion 170 um), s/p EMR on 2019/12/24, s/p laparoscopic AR on 2020/11/20
  2. HP infection s/p eradication (20+ years ago)

主訴

- for scheduled operation

病史

- This is a 57 y/o man with history of HP infection s/p eradication (20+ years ago). He had ever s/p EMR on 2019/12/24 which revealed S Colon Cancer, cT1NxM0 (SM invasion 170 um), (PET: no distant metastasis sign). He was then referred to our CRS OPD and colonoscope was arranged and malignancy couldn't be r/o so operation was suggested for him. BWL was noted in recent days. This time, he is admitted for operation.

體檢發現

- 20191224 Colonoscopy
  Adenocarcinoma, moderately differentiated, arising from tubulovillous adenoma. patho with SM invasion, The depth of invasion is around 170 um below the muscularis mucosa.
  - General appearance :
    - easy looking , conscious clear , without cardiopulmonary distress
    - Vital signs自動帶入date 體溫(BT) 脈搏(P) 呼吸(R) 血壓(BP) 身高(BH) 體重(BW) BMI
      20201119 37℃ 98次/分 18次/分 103/74mmHg 160.2cm 47.8kg 18.6
  - HEENT :
    - Eyes: no anemic conjunctiva, no icteric sclera, normal EOM
    - ENT: no otorrhea, no rhinorrhea, no erythematous mucosa, no injected tonsil, no deformity
  - Neck: supple, freely movable, trachea midline,no deformity, no engorged jugular vein , no palpable thyroid or lymph node enlargement
  - Chest:
    - Inspection: Grossly normal, symmetric and fully expansion, no accessory muscle used, no operation scar, no spider angiomata, no gynecomastia
    - Palpation: no palpable mass or nodule, no subcutaneous emphysema, no tenderness
    - Ausculation: clear breathing sound
  - Heart:
    - Percussion: normal heart size
    - Ausculation: regular heart beat, normal S1, S2, no gallop, no murmur
  - Abdomen:
    - Inspection: in
      - flat contour, symetrically, no scar, no stria, no dilated veins, no rashes
    - Ausculation: normoactive bowel sound, no abdominal bruits

- palpation: soft, no muscle guarding or rigidity, no local tenderness, no rebounding pain, no palpable mass, no palpable pulsation
- Percussion: no shifting dulllness, no hepatomegaly, no splenomagaly

Peripheral pulsation :	CA	BA	RA	FA	PA	PTA	DPA
Right side :	++	++	++	++	++	++	++/+++
Left side :	++	++	++	++	++	++	++/+++

- Genito-anus: no tenderness or palpable mass lesion or rectal shelf, normal external genitalia, normal anal tone, brown stool

CRS CONDITION

- Lung

手術日期及方法

- 手術日期及方法20201120 1. Laparoscopic anterior resection with end-to-end anastomosis, endo-GIA 60/4.1mm*1, ILS 33mm, re-inforcement with silk: nil, bilateral doughnuts: intact, air leak test: done, no air leak.
  2. Low ligation (+): IMA below left colic artery.
  3. Cut margin: unable to see scar of polypectomy,
  4. Reperitonealization: nil.
  5. Take down of splenic flexure: yes.

住院治療經過

- After admission, operation was done on 11/20. Try clear liquid diet on 11/20. Try semiliquid diet on 11/23. Try soft diet on 11/24. Due to relative stable condition, MBD was permitted and OPD f/u is scheudled for further treatment.

合併症

一般檢查

特殊檢查

病理報告

其它

出院時情況

- 15.計劃性出院

出院指示

藥名	劑量	劑量單位	用法	頻次	數量	數量單位	現況	天數	單位
(□)Takepron OD tab 30mg	1	TAB	PO	QDAC	7	TAB	TH	7	天
Ultracet tab	1	TAB	PO	Q6H	28	TAB	TH	7	天
$Biofermin tab	2	TAB	PO	TID	42	TAB	TH	7	天

出院計劃

主治醫師 2062F 蔣鋒帆 / 住院醫師 2310K 陳瑲夫

正式摘要(**電子簽章：000000203924837)**

W55 / W55

doctorRank:B
